# Optical Properties of Single Layer Cu_2_WSe_4_ from the *Ab Initio* Bethe–Salpeter Equation Method

**DOI:** 10.1021/acs.jpcc.5c00855

**Published:** 2025-04-22

**Authors:** Tarik Ouahrani, A. Esquembre Kučukalić, R. M. Boufatah, Daniel Errandonea

**Affiliations:** † École Supérieure en Sciences Appliquées, ESSA-Tlemcen, BB 165 RP Bel Horizon, Tlemcen 13000, Algeria; ‡ Laboratoire de Physique Théorique, Université de Tlemcen, BP 119, Tlemcen 13000, Algeria; § Departamento de Física Aplicada - Institute of Materials Science (ICMUV), University of Valencia, Catedrático Beltrán 2, Valencia E-46980, Spain; ∥ Matter at High Pressure (MALTA) Consolider Team, Universidad de Valencia, Edificio de Investigación, C/Dr. Moliner 50, Burjassot, Valencia 46100, Spain

## Abstract

The binding energy of excitons is essential in assessing the suitability of materials for photovoltaic applications. This research employs first-principles calculations based on the *GW* approximation and the Bethe–Salpeter equation to explore the excitonic characteristics of a Cu_2_WSe_4_ monolayer. Our findings support the structural stability of this two-dimensional material and demonstrate a pronounced excitonic response. The computed binding energies for both bright and dark excitons are considerably larger than those that are generally necessary for standard photovoltaic applications. However, examination of exciton amplitude reveals a highly delocalized configuration of electron–hole pairs throughout the crystal, which may alleviate some issues related to elevated binding energies. These results highlight the excitonic properties of Cu_2_WSe_4_ and offer valuable insights into its potential for optoelectronic applications.

## Graphical abstract

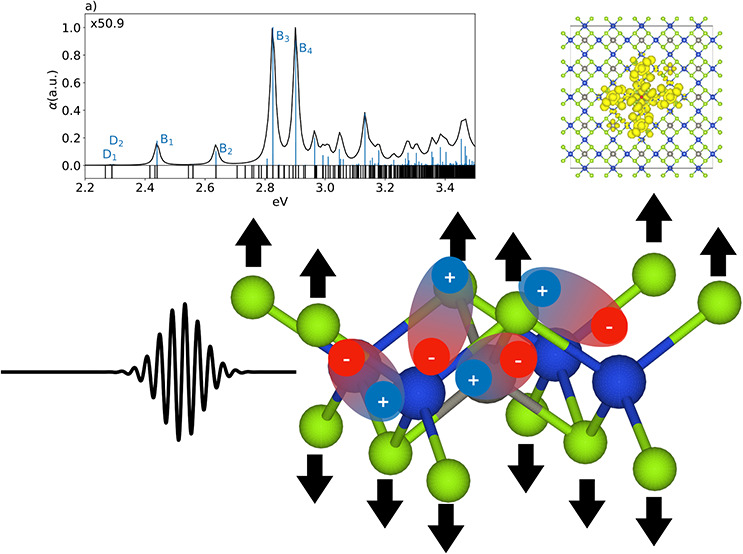

## Introduction

An important factor influencing the conversion of sunlight into electricity in photovoltaic (PV) systems is its close relationship to exciton binding energy, which significantly affects the efficiency of charge carrier generation and separation.^1^ The energy required to free the bound electron–hole pair (exciton) into free carriers is commonly referred to as the exciton binding energy (*E*_*b*_). A low *E*_*b*_ allows excitons to dissociate into free carriers through thermal agitation or weak external fields, thus facilitating the generation of free charge carriers.^2^ In contrast, tightly bound excitons with large *E*_*b*_ (e.g., > 100 meV)^1^ are less likely to dissociate into free carriers under standard conditions. As a result, excitons decay back to the ground state without producing free carriers, leading to high nonradiative recombination rates and reduced photocurrent generation.^3^

Because of the unique characteristics of two-dimensional (2D) structures, a significant body of research has focused on exploring their potential for use in photovoltaic applications. These structures include the transition metal dichalcogenide (TMD) family, IV-group monochalcogenides,^4,5^ and related subclasses.^6^ 2D van der Waals heterostructures^7^ provide a fresh perspective on fundamental exciton physics. In semiconducting 2D materials, excitonic effects significantly influence their optical characteristics, primarily as a result of decreased dielectric screening and the confinement of charge.^8^ This results in exceptionally strong Coulomb interactions, leading to many-particle phenomena, such as the formation of various exciton types, including optically allowed bright excitons, optically forbidden dark excitons, and spatially separated interlayer excitons.^9^ Furthermore, inorganic 2D materials often exhibit long carrier lifetimes and high mobility, which are crucial for efficient charge transport and reduced recombination losses.^10^ The ability of these materials to be engineered at the atomic scale opens new opportunities for designing efficient, flexible, and lightweight solar cells.^11^

Cu_2_WX_4_ ternary materials based on chalcogenide atoms (X) have emerged as one of the most extensively researched 2D materials for a variety of uses.^12^ In particular, they are widely used in the photocatalytic (PTC) field.^13^ As in the case of photovoltaic mechanisms, a high exciton binding energy is usually seen as a sign of a strong Coulombic interaction between electrons and holes, which makes it difficult for visible or ultraviolet (UV) light to be absorbed. However, for photocatalytic water splitting, large exciton binding energy is generally unfavorable unless effective charge-separation mechanisms mitigate it. This also highlights the importance of moderate energy binding in PV and PTC applications. In fact, Cu_2_WS_4_ has shown drawbacks as an efficient PTC material, and to be more efficient, the structure of the compound must be tuned either by applying an external field or by defect engineering.^13^ A recent study^14^ suggested that the pristine structures of Cu_2_WSe_4_ possess an efficient absorption coefficient. The authors calculated the photocurrent in Cu_2_WSe_4_-based nanodevices and proposed Cu_2_WSe_4_ as a promising photovoltaic material. However, the study does not include any evaluations of the level of excitonic binding. In addition, the localization of excitons is another problem that deserves careful examination. The intrinsic electric field or interfacial forces in a photovoltaic device find it challenging to separate the exciton into free carriers because the electron and hole are confined to a small area.^15^

In this work, we explore the excitonic properties of single layers of Cu_2_WSe_4_ using a many-body perturbation theory. The Bethe-Salpeter equation (BSE) is solved on top of a single-shot *GW*-corrected band structure (*G*_0_ *W*_0_). The advantage of the BSE+*GW* method is double. First, the *GW* band structure accounts for screening effects not present in the Kohn–Sham theory. Second, BSE provides a direct method for studying the excitonic wave functions in real and reciprocal space, permitting the analysis of electron–hole localization. The extraction of the exciton spectrum from the BSE yields a straightforward computation of the binding energy of each exciton as the difference between the *GW* gap and its proper energy. We, therefore, report the BSE+*GW* analysis of Cu_2_WSe_4_ to describe its excitons as highly binding with a rather small degree of crystal localization.

## Methods

In this study, first-principles calculations were performed using Density Functional Theory (DFT),^16^ as implemented in the Quantum ESPRESSO code (QE).^17,18^ Two steps were followed. First, a relaxation of the unit cell and atomic positions was conducted utilizing the generalized-gradient approximation (GGA) along with the Perdew–Burke–Ernzerhof (PBE) parametrization^19^ for the exchange-correlation interaction, consistent with methodologies previously reported in the literature.^20^ In this process, to account for long-range van der Waals interactions in the crystal structure, the DFT-D2 method proposed by Grimme^21^ was employed. A vacuum space of 22.6 Å along the **z**-axis was included to avoid interactions between adjacent virtual layers. A plane-wave cutoff of 60 Ry and an 8 × 8 × 1 Monkhorst–Pack **k**-point sample of the Brillouin zone^22^ were used in the calculations of the ground-state density. The atomic forces converged to a maximum value of 10^–5^ Ry/bohr, and the geometries were relaxed until the total energy converged to 10^–4^ Ry. Second, for the single-shot *GW* correction^23^ and the examination of the optical and excitonic properties, the YAMBO code was employed.^24^ In this step, the Kohn–Sham band structure was used as a starting point in calculating excited properties with the Bethe-Salpeter framework. We converged the self-consistent cycle with a plane-wave cutoff of 150 Ry and a k-mesh of 12 × 12 × 1. Convergence tests of excited-state properties are gathered in the appendix to this paper. To check the dynamical stability of the studied structure, phonon dispersion calculations were performed using the Phonopy package^25^ with a 6 × 6 × 1 k-mesh and a 2 × 2 × 1 supercell.

## Results and Discussion

### Stability of the Cu_2_WSe_4_ Single Layer

As shown in Figure 1, the single-layer Cu_2_WSe_4_ is made up of a 2D crystal lattice with atoms of copper (Cu), tungsten (W), and selenium (Se) arranged periodically. The spatial arrangement and bonding within the layer are highlighted by the three projections of the structure. The structure has a high in-plane symmetry. Each Cu atom is surrounded by Se atoms, forming a distorted octahedral coordination. The W atoms are tetrahedrally coordinated by Se atoms, with W–Se bonds providing structural rigidity. The convex hull diagram can act as a foundation for providing critical insight into the thermodynamic stability of 2D Cu_2_WSe_4_. We plot a 3D representation of these diagrams in Figures S1–S4. The stability of Cu_2_WSe_4_ is indicated by its position on the hull, representing a minimum in Gibbs free energy relative to other possible phases in the system. The competing stable phases surrounding Cu_2_WSe_4_ include WSe_3_, Se_2_Cu_2_, Se_2_Cu_3_, and the elements tungsten (W), copper (Cu), or selenium (Se). These competing phases define the boundaries of the stability region of Cu_2_WSe_4_, meaning that any deviation in the chemical potential of W, Cu, or Se beyond this range could lead to decomposition. For instance, under Se-rich conditions, Cu_2_WSe_4_ may decompose into WSe_3_ and a copper selenide such as Cu_2_Se_2_ or Cu_3_Se_2_. Similarly, Cu-rich conditions may favor the formation of segregated Cu alongside WSe_3_, while W-rich environments could stabilize isolated W and selenium-rich compounds. Cu_2_WSe_4_ on the convex hull confirms its thermodynamic stability within a specific range of chemical potentials, emphasizing the importance of maintaining precise synthesis conditions to avoid phase decomposition into the competing phases.

**Figure 1:**
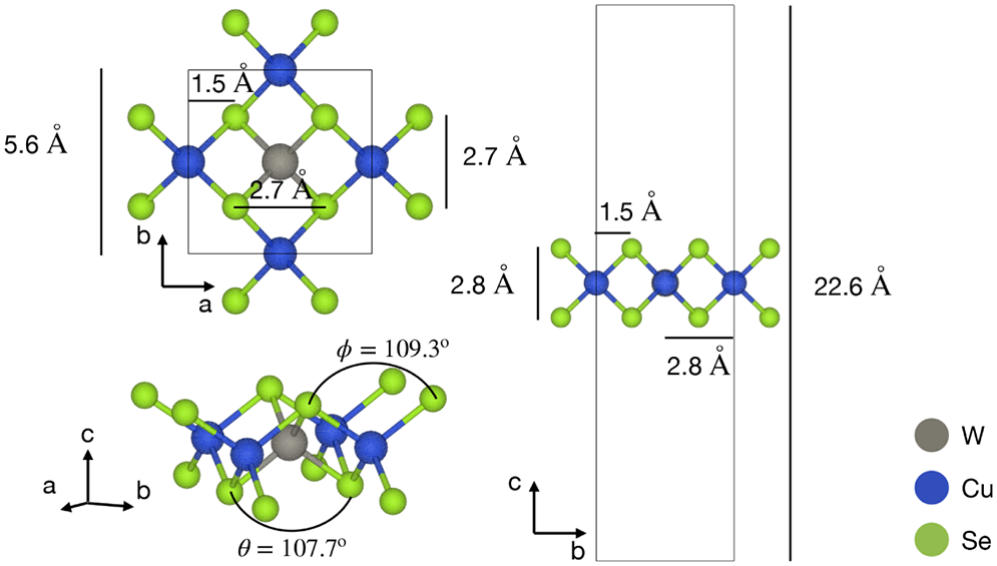
Top, side, and perspective views of Cu_2_WSe_4_ monolayer. The Cu and W atoms are at the same height at the middle of the *c* axis, between two equidistant layers of Se atoms.

We continue by analyzing the stability of the examined structure. First, we optimized the crystal structure. The computed PBE + D2 lattice parameter (5.61 Å) agrees with the 5.60 Å value of recent theoretical calculations of ref 14. For the optimized Cu_2_WSe_4_ single layer, the phonon dispersion, elastic constants, and formation energy were also calculated. The phonon dispersion is shown in Figure 2. The lack of imaginary branches indicates the dynamic stability of 2D Cu_2_WSe_4_. Our phonon dispersion is consistent with previously reported results for similar materials such as Cu_2_WS_4_,^13^ Cu_2_MoS_4_,^26^ and a similar crystal structure has already been published in ref 14. The acoustic modes exhibit smooth behavior and linear dispersion near the Γ point, as expected for a mechanically stable structure. Three acoustic and 18 optical modes make up the 21 branches in the phonon dispersion. A point group symmetry analysis of the modes at the center of the Brillouin zone yields Γ = 2*A*1 + 2*A*2 + *B*1 + 4*B*2 + 6*E*, where Γ*acoustic* = *B*_2_ + *E* and Γ*optic* = 2*A*_1_ + 2*A*_2_ + *B*_1_ + 3*B*_2_ + 5*E*. The 2*A*_1_ + *B*_1_ + 3*B*_2_ + 5*E* modes are Raman-active, while the 3*B*_2_ + 5*E* modes are also infrared-active. The *A*_2_ modes are silent. Table 1 provides a summary of the computed wavenumber, symmetry, and activity for each phonon. There are no experimental results to compare with, and previous calculations^14^ did not report phonon wavenumbers. The optical branches are isolated into different frequency bands, reflecting distinct vibrations involving Cu, W, and Se atoms. The Se contributions are dominantly observed at higher frequency ranges, which is consistent with a light mass and a strong bonding, whereas the Cu contributions are found across a wide range of frequencies, contributing to both low- and midfrequency vibrational modes. Finally, the W contributions are primarily concentrated in the low-frequency region due to its higher atomic mass.

**Figure 2:**
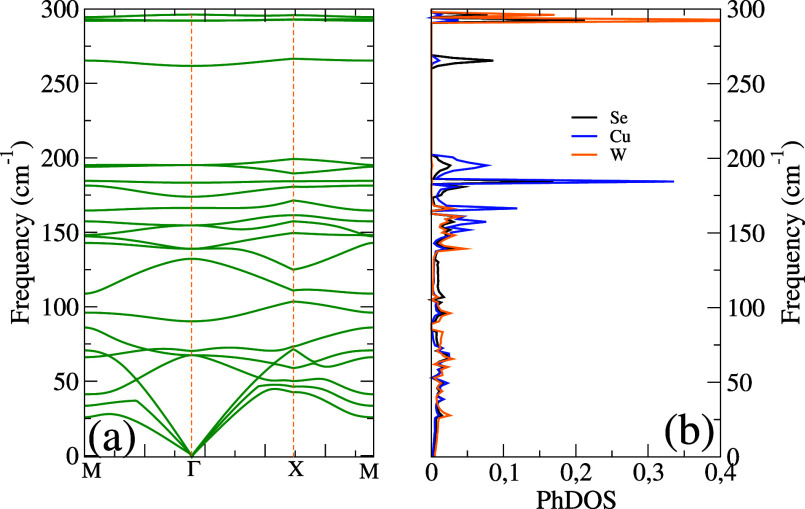
(a) The phonon dispersion and (b) the corresponding phonon density of states (PHDOS) of the Cu_2_WSe_4_ monolayer.

**Table 1:** Calculated Modes of 2D Cu_2_WSe_4_ ^a^

Symmetry	ω (cm^–1^)	Activity
E	67.4843	R-IR
A_2_	70.3731	S
B_2_	90.2497	R-IR
A_1_	132.217	R
E	138.931	R-IR
E	154.705	R-IR
B_2_	166.416	R-IR
B_1_	173.907	R
A_2_	183.317	S
E	195.141	R-IR
A_1_	261.734	R
E	292.123	R-IR
B_2_	296.162	R-IR

a Raman (R), infrared (IR), and silent (S) modes are indicated.

The elastic stiffness components were used to obtain the mechanical properties of 2D Cu_2_WSe_4_ using the stress–strain relation. For comparison, Table 2 provides the C_11_, *C*_12_ and C_66_ = *G* components compared with the same parameters of Cu_2_WS_4_, G being the shear modulus. The elastic constants meet the Born-Huang (C_11_ > 0 and C_11_ – C_12_ > 0) requirements,^27^ which indicates that the monolayer of Cu_2_WSe_4_ is mechanically stable. In comparison to Cu_2_WS_4_, we can observe that Cu_2_WSe_4_ has a lower in-plane stiffness. This implies that Cu_2_WS_4_ has greater resistance to uniaxial strain along the in-plane directions and has a stiffer mechanical structure. Compared to Cu_2_WS_4_ (29.59 N/m), the *C*_12_ value for Cu_2_WSe_4_ (4.755 N/m) is substantially smaller. This suggests that Cu_2_WSe_4_ is less anisotropic than Cu_2_WS_4_ because it shows a weaker coupling between strains along perpendicular directions. Compared to Cu_2_WS_4_ (G = 9.02 N/m), Cu_2_WSe_4_ (G = 2.323 N/m) has a substantially smaller shear modulus. The softer bonding framework brought about by the heavier Se atoms is probably the reason for this lower value, which indicates that Cu_2_WSe_4_ is less resistant to shear deformations. Consequently, the overall rigidity of the lattice is decreased when S is replaced with Se, which may have an impact on the suitability of the material for uses like strain engineering or mechanical flexibility in device design.^28,29^

**Table 2:** Calculated Elastic Constants C_ij_ of Single-Layered Cu_2_WSe_4_ ^a^

	C_11_ (N/m)	C_12_ (N/m)	G (N/m)
Cu_2_WSe_4_	40.604	4.755	2.323
Cu_2_WS_4_	51.83	29.59	9.02

a The table also gives the shear modulus (G).

### Optical Properties

To investigate the optical properties of Cu_2_WSe_4_, we start by correcting the Kohn–Sham band structure with the single-shot *GW* approximation (see Appendix A). The result is shown in Figure 3. We observe that the conduction bands in the *GW* approximation are shifted by ∼2 eV upward in comparison to those at the independent-particle limit (DFT). Such behavior is expected from the *GW* correction of the Kohn–Sham (KS) band structure, which is related to the inclusion of dynamical screening effects on the Coulomb interaction between excited electrons and valence charges.^10^ The valence bands in this plot seem to move slightly downward when quasiparticle (QP) effects are included, with a little stretching and the formation of a continuum in the occupied energy levels. The small steepening present in the *G*_0_ *W*_0_ bands suggests potential changes in carrier mobility. The curvature of the highest unoccupied states, which is related to the effective mass of charge carriers, appears to be consistent between KS and *G*_0_ *W*_0_. The direct gap in KS is 1.62 eV at a point between X and M, while the indirect gap is 1.26 eV, connecting M and Γ. The direct gap in *GW* is 3.80 eV at M, and the indirect gap is 3.53 eV, between M and X.

**Figure 3:**
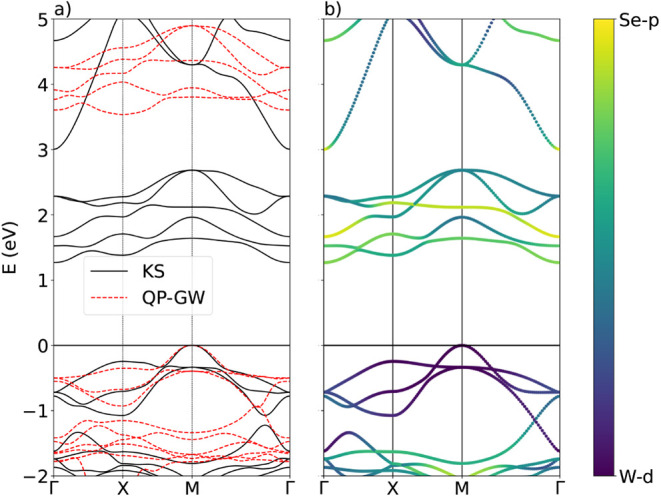
(a) DFT (KS) and *GW* (QP) band structure of Cu_2_WSe_4_. (b) Projection of the Kohn–Sham band structure in *d* orbitals of the W atom and *p* orbitals of the Se.

The orbital projection of the band structure is shown in Figure 3b, on top of the KS energy levels (see also Figure S5). We compare the contribution from *d* orbitals of the W atom and *p* orbitals of the Se atom since they are the main source of charge carriers. Close to the Fermi level, valence bands have a high contribution of W-*d* orbitals, while conduction bands are slightly hybridized with Se-*p* orbitals. This means that low-energy transitions are expected to happen between W-*d* and Se-*p* orbitals. In particular, since excitons are bound states between an excited electron and a positively charged hole in the valence bands, it is anticipated that the lowest-energy electron–hole pairs are localized around W-*d* and Se-*p* orbitals.

To obtain a precise response to this prediction, we solve the Bethe-Salpeter equation. In the BSE framework, each exciton is made up of a linear superposition of transitions from occupied bands *v* to empty bands *c* at crystal momentum **k**, as in eq 16 of Appendix B. Each possible linear combination is an eigenstate of the Bethe-Salpeter Hamiltonian in eq 14, which allows for an energy labeling of the excitons. The excitonic energies are the poles of the polarizability α, represented in Figure 4. The figure presents the absorption spectrum of Cu_2_WSe_4_ under quasi-particle corrections (QP). Below the spectrum, eigenvalues are also shown as vertical black lines. Excitons are considered as dark (D_*i*_) or bright (B_*i*_) depending on their oscillator strength below or above 0.1, respectively. Dark excitons are optically inactive but can influence nonradiative recombination and other processes. These effects are critical for understanding the optical properties of Cu_2_WSe_4_ and are not captured in the independent particle (IP) approximation of the KS theory. The QP spectrum demonstrates strong excitonic effects, as evidenced by the pronounced peaks and large oscillator strengths for B_1_, B_2_, and B_3_. The presence of dark excitons (e.g., D_1_) below the first bright exciton (B_1_) is characteristic of many 2D semiconductors.

**Figure 4:**
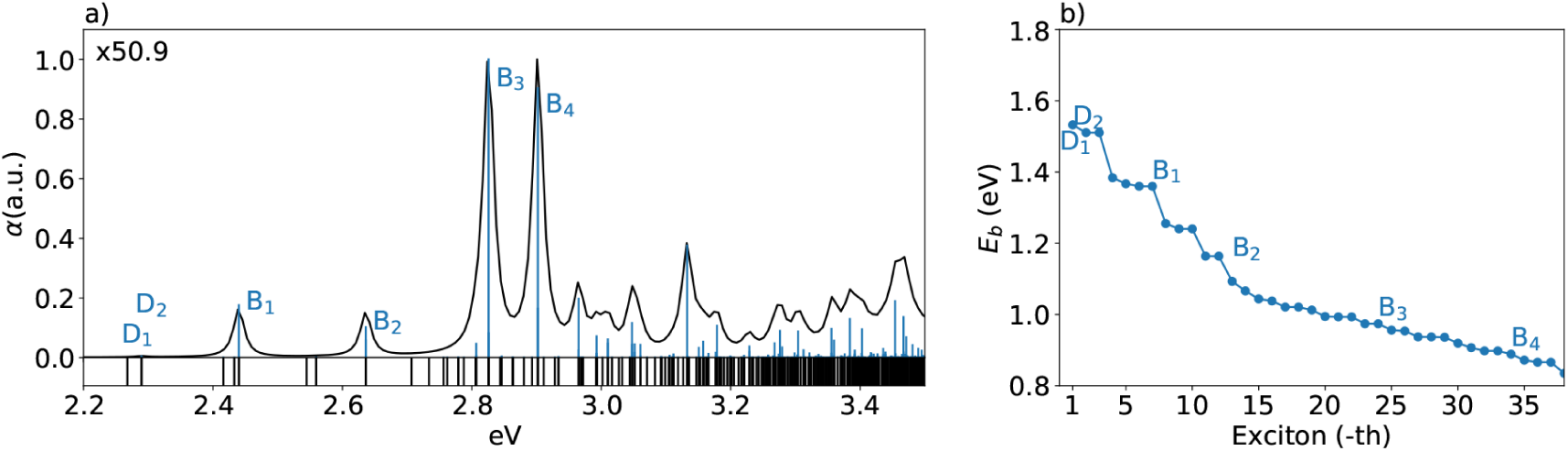
(a) The absorption spectrum of Cu_2_WSe_4_ in atomic units with quasi-particle corrections. Below, the eigenvalues are shown as vertical black lines. Some dark excitons are labeled as D_*i*_, while bright excitons, as B_*i*_, according to their oscillator strength, are represented as blue lines normalized to the absorption maximum, which corresponds to the bright exciton B_4_. (b) Exciton binding energies are calculated as the energy difference between the *GW* gap and each exciton energy.

Assuming the *G*_0_ *W*_0_ gap $E_{g}^{GW}$ is the fundamental gap of the system, each electron–hole state *i* has a binding energy $E_{b}^{i}=E_{g}^{GW}$ – *E*_*i*_, where *E*_*i*_ is its corresponding pole energy. From the excitonic spectrum in Figure 4a, the binding energies of some excitons are plotted in Figure 4b. Binding energies decrease as the exciton energy increases. However, for low-energy excitons in Cu_2_WSe_4_, the binding energy reaches a maximum of approximately 1.6 eV, an order of magnitude higher than the 100 meV lower threshold typically required for effective photovoltaic performance.^2^ Low-energy excitations, which can be generated from the absorption of long-wavelength radiation, expand the usable portion of the solar spectrum. However, in this system, they have high binding energy, which is a handicap in the possibility of electron–hole dissociation into free charge carriers. Since photovoltaic applications require free charge carriers in the generation of current, the values of the binding energies for Cu_2_WSe_4_ might make it rather an efficient radiation absorber. This implies a possible lack of enough capabilities for photocurrent applications.

The BSE formalism also allows for the study of excitonic localization. We distinguish between localization in the reciprocal space, and in real space. Namely, in the transition basis (see Appendix B), the quantum superposition of vertical transitions may involve only a subset of points in the Brillouin zone and a specific number of bands. The smaller these two numbers are, the higher the reciprocal-space localization is. For real-space localization, a hole is assumed to be located at some specific position. Then, the exciton amplitude is studied as a function of the electronic position. This provides the likelihood of, having spotted a hole, finding its bounded electron elsewhere. The radius around the hole position in which the excitonic amplitude is big informs about excitonic localization in real space. For a huge radius, a fixed hole interacts with an electron at any position through the crystal, implying a big excitonic delocalization. Since real and reciprocal spaces are connected by a Fourier transform, real-space localization indicates a possible reciprocal-space delocalization, and vice versa.

This is illustrated in Figure 5 for the first dark exciton, D_1_ (a), and the brightest exciton, B_4_ (b). D_1_ is primarily composed of vertical transitions close to the Fermi level around *M*, with negligible contributions from other regions of the Brillouin zone. This denotes strong localization in reciprocal space, and that its real-space localization might be rather high. In fact, the excitonic probability distribution in the crystal is also depicted in Figure 5. It is shown as an isosurface region of space where the squared amplitude is constant. By normalizing the amplitude to its maximum value, the shown isosurface highlights areas where the probability distribution reaches 1% of the maximum value. When a hole is assumed to be on top of a W atom, the probability distribution of electrons shows hoppings to neighboring atoms. Similarly, if the hole is located in a Se-*p* orbital, the results confirm the same behavior, as shown in the lower panels of Figure 5a. We infer that the maximum amplitude is localized around the first neighboring atoms and that the spatial localization is quantified by a hopping probability to first-neighboring atoms of 1% of the maximal amplitude.

**Figure 5:**
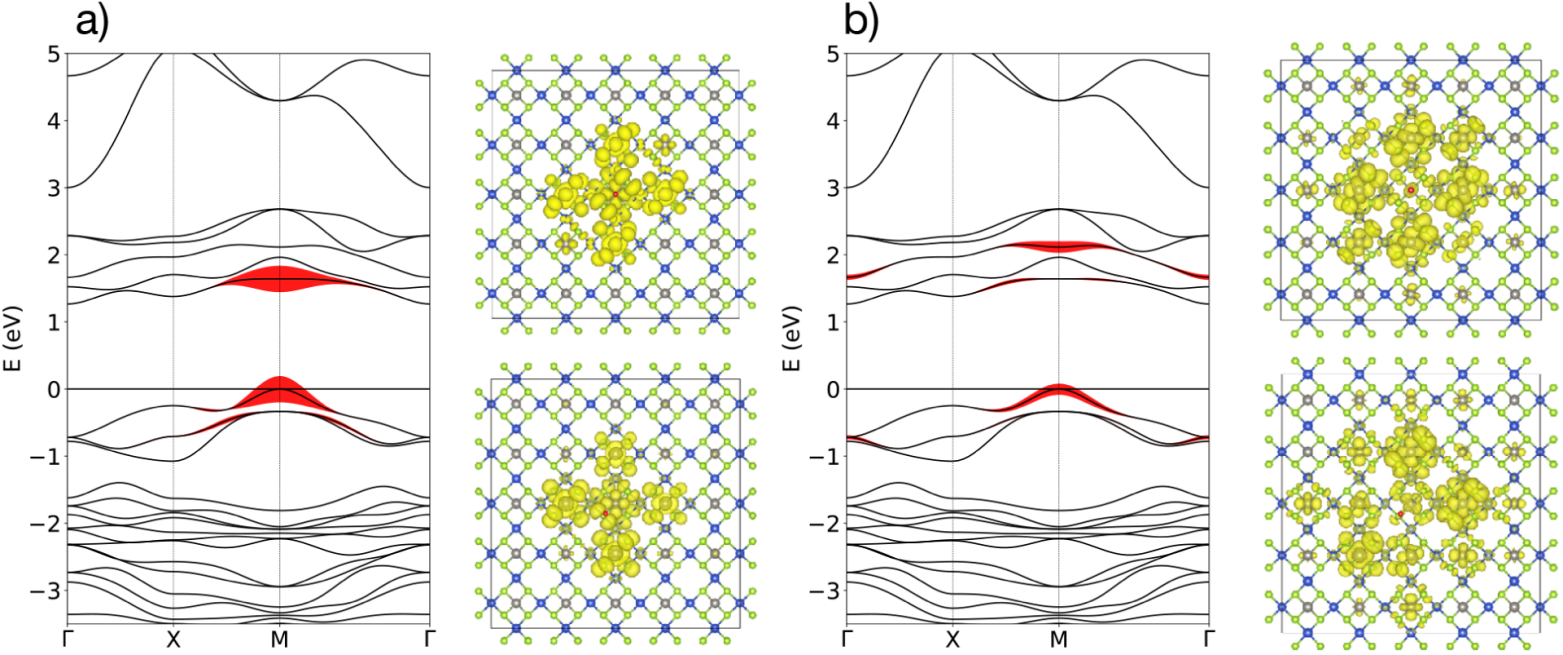
Excitonic amplitudes in transition space and in real space. (a) First dark exciton, D_1_. (b) Brightest exciton, B_4_. Holes on top of the W atoms (top) and next to Se atoms (bottom). The isosurface of each plot has been tuned manually to visualize similar features. A red circle indicates the position of the hole. Isosurface: 1% on from zero value.

The brightest exciton B_4_ is analyzed in Figure 5b. Here, a slightly higher degree of reciprocal-space delocalization is present, since transitions at *M*, Γ and between *X*-*M* contribute to the excitonic wave function of eq 16. Indeed, for a We-*d* hole, only at 1% of the maximum amplitude spatial delocalization starts to be visible. This means that the likelihood for a W-*d* valence electron to hop into a conduction orbital elsewhere in the crystal is also small, but bigger than for D_1_. Yet, we see some hopping probability to second neighboring cells not present in the isosurface of D_1_.

According to Figure 4, the bright exciton energy is close to the direct band gap energy and, from Figure 5, it is localized around the M-X and *M*-Γ directions. This concentration of transitions at symmetry points might affect its mobility and lifetime. The dominance of transitions near M suggests this point is critical for tailoring optical and electronic properties through external factors like strain or electric fields. In addition, these localized transitions align with the selection rules for light absorption. To provide additional context, Figure S5 shows the projected density of states (DOS) for Cu_2_WSe_4_. This reveals that excitonic transitions predominantly involve contributions from Cu/W-*d* and Se-*p* orbitals., as expected from Figure 3b.

## Conclusions

In conclusion, although the existing literature highlights the potential of Cu_2_WSe_4_ for photovoltaic applications, its applicability is hindered by certain drawbacks. The high exciton binding energies, primarily resulting from the approximately 4 eV fundamental gap, present a considerable barrier to effective electron–hole dissociation, which is essential for the generation of free charge carriers in photovoltaic devices. Furthermore, the delocalization of both dark and bright excitons throughout the crystal indicates that charge transport may be ineffective, as only a limited number of unit cells contribute to the excitonic states. Nevertheless, the localization in reciprocal space offers insights that could facilitate exciton dissociation through external influences such as applied electric fields, strain engineering, or doping, which may reduce the binding energy or improve carrier separation. Consequently, while direct applications in photovoltaic applications may be constrained, Cu_2_WSe_4_ possesses characteristics that warrant further investigation into its potential for radiation absorption or other optoelectronic applications.

## Supplementary Material



## Figures and Tables

**Figure 6:**
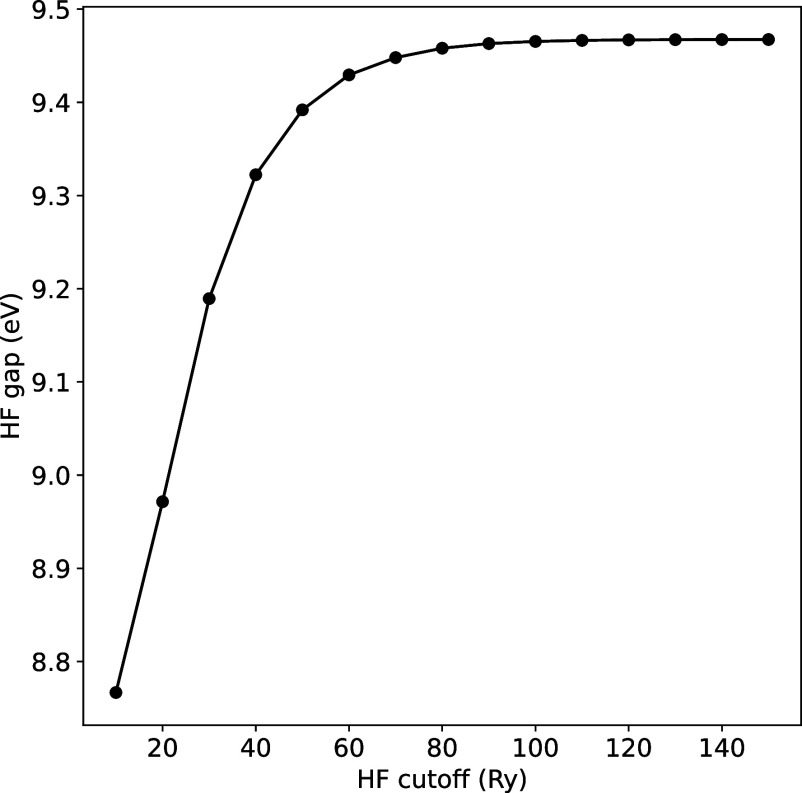
Convergence of the reciprocal-space sum in the Hartre-Fock exchange. Computed from a Kohn–Sham band structure employing a 4 × 4 k-mesh.

**Figure 7:**
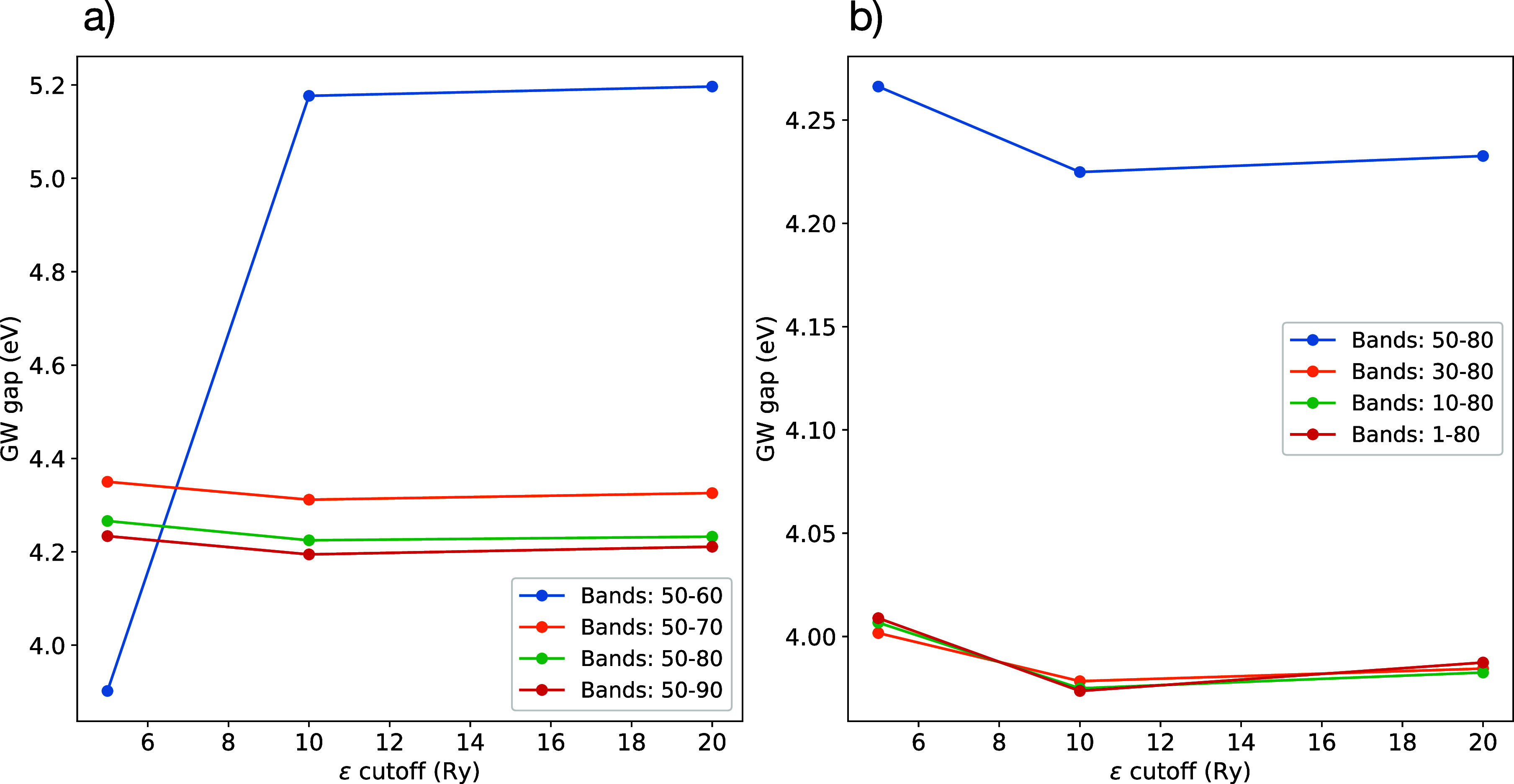
Convergence of the *GW* gap as a function of the dielectric function parameters. (a) Variation on the conduction bands. (b) Variation on the valence bands.

**Figure 8:**
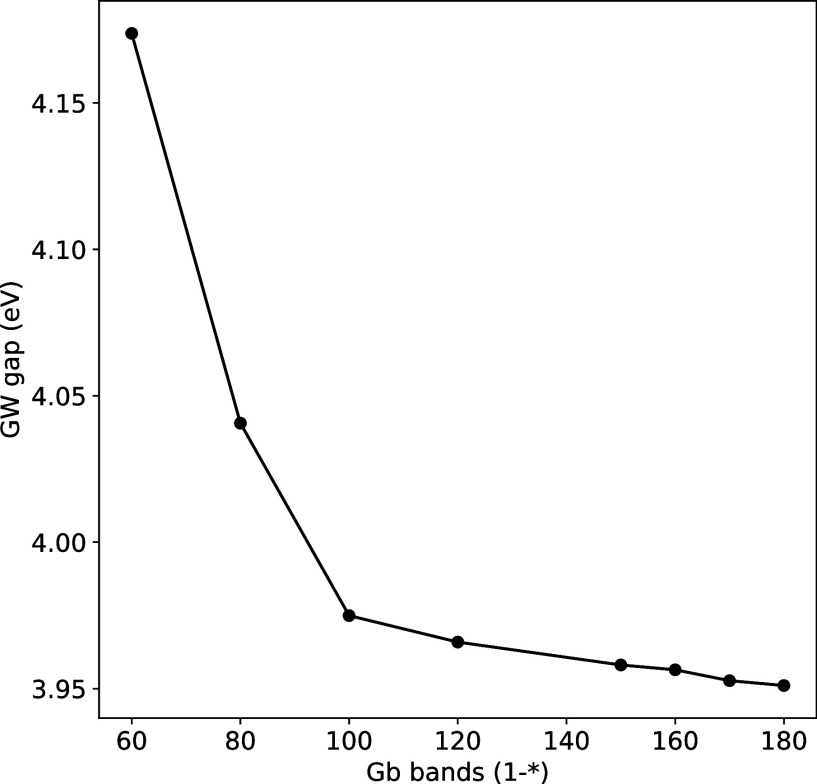
Convergence of the correlation self-energy.

**Figure 9:**
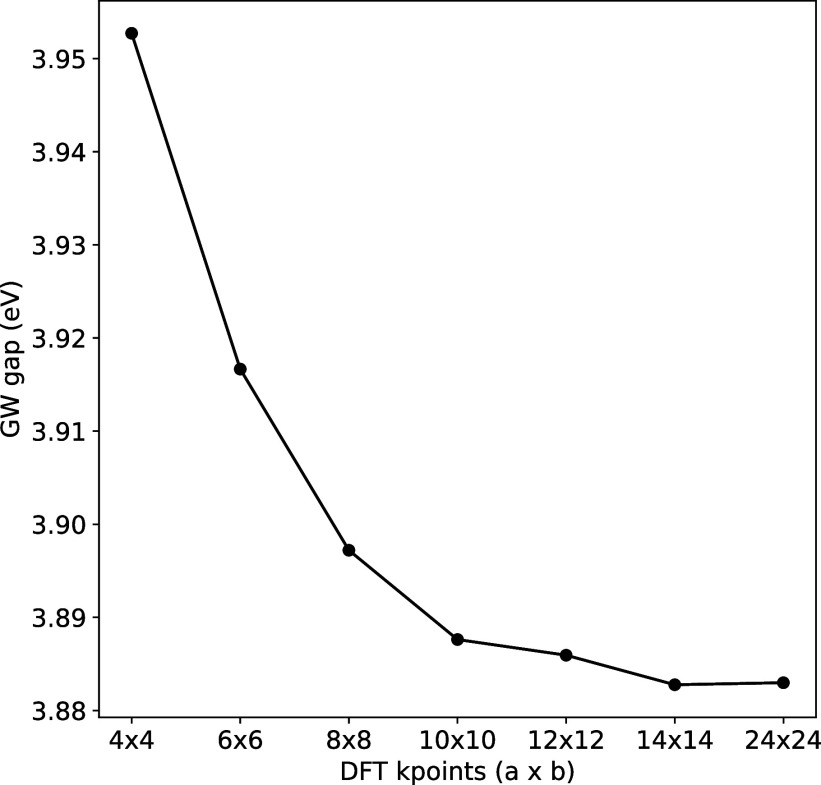
Convergence of the *GW* gap with the sample of the Brillouin zone.

**Figure 10:**
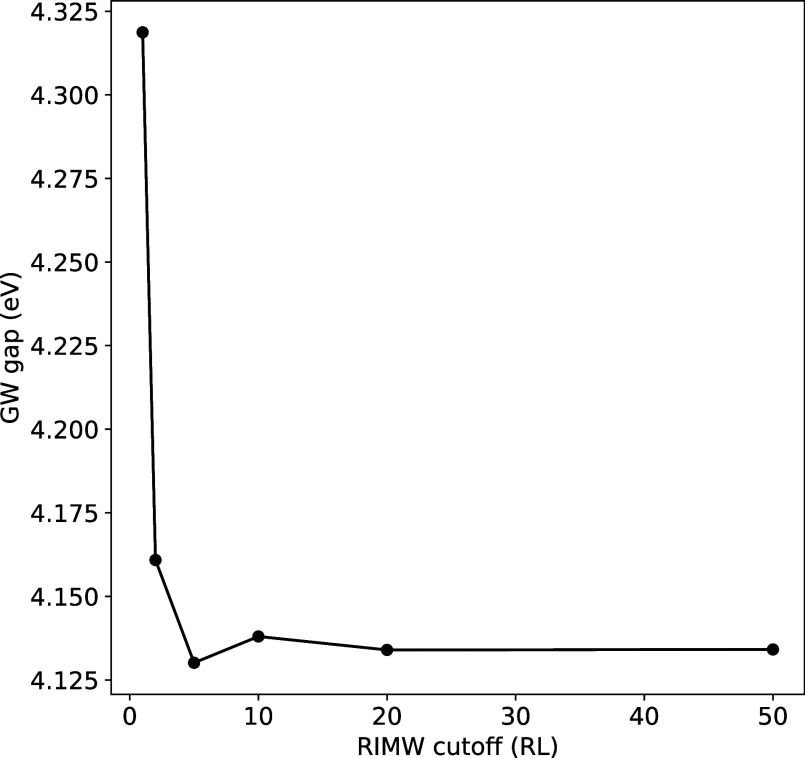
Convergence of the *GW* gap with the sample of the Brillouin zone from the stochastic integration of the screened potential.^34^ This test was performed on a 4 × 4 × 1 Monkhorst–Pack mesh, with 90 Ry in the Hartree–Fock cutoff, a screening of 10 Ry and 10 bands, and 100 bands in the correlation self-energy. RL indicates the number of reciprocal lattice vectors in which the stochastic correction of the screened potential is applied.

**Figure 11:**
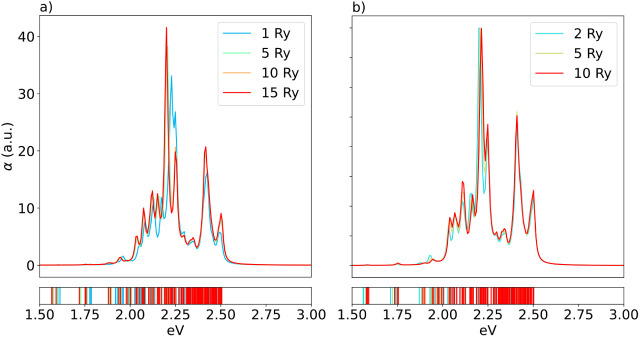
Convergence of the BS spectrum with the cutoff of the dielectric function.

**Figure 12:**
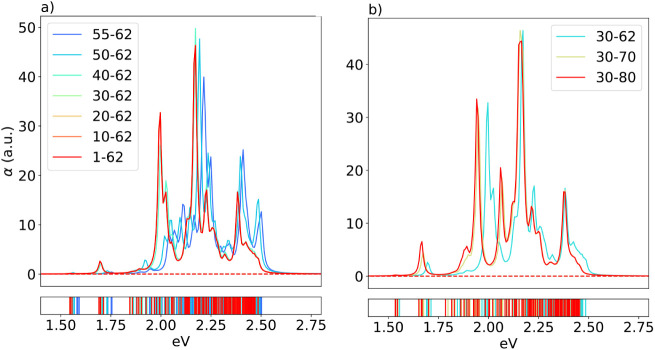
Convergence of the BS spectrum with the number of bands in the construction of the Kernel. (a) Convergence of the valence bands. (b) Convergence of the conduction bands. The gap between the 58th and 59th bands.

**Figure 13:**
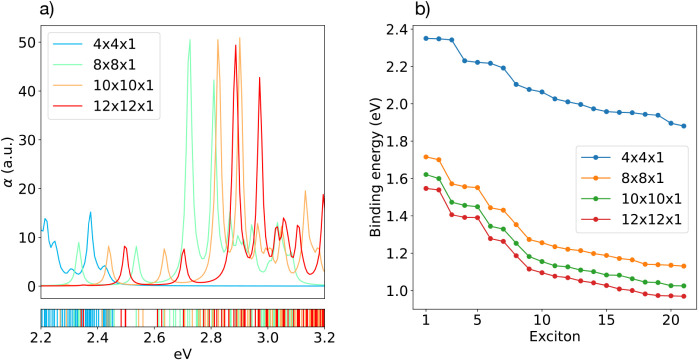
Convergence of the BS spectrum (a) and the binding energies (b) with the sampling of the Brillouin zone. For the panel b), the binding energies have been calculated with the 10 × 10 × 1 *GW* gap.

## Appendix A

### The *GW* Band Structure

The DFT band structure does not yield reliable values for the electronic gap. The *GW* corrections provide accurate improvements to the DFT electronic structure within the Kohn-Sham framework.^23^ First, if the electronic wavefunction is known to correspond to the fully interactive many-body Hamiltonian, Green’s function accounts for the transition likelihood of an electron under all possible interactions:^30^

1
$$G\left(r_{1},t_{1};r_{2},t_{2}\right)\equiv \frac{1}{i\hbar }\left\langle GS\right|T\psi \left(r_{1},t_{1}\right)\psi^{†}\left(r_{2},t_{2}\right)\left|GS\right\rangle$$

where |*GS*⟩ represents some ground state and time ordering is assumed. In practice, the computation of *G* goes through a cycle of five correlated magnitudes according to Hedin’s equations.^31^ Namely, Green’s functions admit a Dyson equation. Employing the notation 1 ≡ **r**_1_, *t*_1_; **r**_2_, *t*_2_, it reads:

2
$$G\left(1,2\right)=G^{0}\left(1,2\right)+\int d3d4G^{0}\Sigma \left(3,4\right)G\left(4,2\right)$$

where *G* ^0^(1,2) is built from independent-particle wavefunctions and Σ­(3,4) is the self-energy, which enters Hedin’s cycle. The so-called *GW* approximation consists of writing the self-energy as a product of Green’s function and the screened Coulomb interaction *W*:

3
$$\Sigma \left(1,2\right)\approx \Sigma^{GW}\left(1,2\right)=\frac{i}{\hbar }G\left(1,2\right)W\left(2,1\right)$$

The result of solving Hedin’s cycle with the *GW* approximation is, in practice, a correction of the Kohn-Sham band structure as^32^

4
Enk≈EnkKS+ZnkRe⟨ψnkKS|Σ(EnkKS)−Vxc|ψnkKS⟩

where $\left\langle \psi_{nk}^{KS}\right|$ are Kohn-Sham eigenstates, and therefore knowledge of the fully-interactive wavefunctions is not needed. *Z*_*n* **k**_ is a normalization factor:

5
Znk=(1−Re⟨ψnk|δΣ(ϵ)δϵ|ϵ=EnkKS|ψnk⟩)−1

and *V*_*xc*_ is the usual DFT exchange-correlation potential. These energies are shown in Figure 3. We solve Hedin’s cycle only once, therefore yielding the single-shot *GW* correction of the band structure (*G*_0_ *W*_0_). Our calculations do not modify the excitonic wavefunctions directly. They are however affected by the *G*_0_ *W*_0_ through the Bethe-Salpeter equation, which is built from the corrected band structure, as outlined in Appendix B.

The self-energy Σ can be separated in its exchange Σ^*x*^ and correlation Σ^*c*^ parts, i.e., $\Sigma_{nk}\left(\omega \right)=\Sigma_{nk}^{x}+\Sigma_{nk}^{c}\left(\omega \right)$ . The exchange part can be obtained from the Hartree-Fock theory:

6
$$\Sigma_{nk}^{x}\equiv \left\langle \psi_{nk}\right|\Sigma^{x}\left|\psi_{nk}\right\rangle =-\underset{m}{\sum }\int_{BZ}\frac{dq}{{\left(2\pi \right)}^{3}}\underset{G}{\sum }v\left(q+G\right)|\rho_{n,m}\left(k,q,G\right){|}^{2}f_{m,k-q}$$

where *v*(**q** + **G**) is the Coulomb interaction, *f*_*m*,**k**‑**q**_ are occupation factors from the DFT band structure, and the dipoles *ρ*_*n*,*m*_(**k**,**q**,**G**) are defined as

7
$$\rho_{n,m}\left(k,q,G\right)=\left\langle \psi_{nk}\right|e^{i\left(q+G\right)\cdot r}\left|\psi_{nk-q}\right\rangle$$

where |*ψ*_*n* **k**‑**q**_⟩ are Kohn-Sham states.

On the other hand, the correlation part requires the integration of the macroscopic dielectric function $\epsilon_{GG\prime }^{-1}$ :

8
$$\begin{array}{c} \Sigma_{nk}^{c}\left(\omega \right)=i\underset{m}{\sum }\int_{BZ}\frac{dq}{{\left(2\pi \right)}^{3}}\underset{GG^{\prime }}{\sum }\frac{4\pi }{|q+G{|}^{2}}\rho_{nm}\left(k,q,G\right)\rho_{nm}^{*}\left(k,q,G^{\prime }\right)\\ \cdot \int d\omega^{\prime }G_{m,k-q}^{0}\left(\omega -\omega^{\prime }\right)\epsilon_{GG^{\prime }}^{-1}\left(q,\omega^{\prime }\right), \end{array}$$

where the dielectric function is calculated in the Plasmon-Pole Approximation,^32^ and $G_{m,k-q}^{0}\left(\omega -\omega^{\prime }\right)$ is the non-interacting Green’s function, calculated from the Kohn-Sham spectrum. With this, the *GW* correction of the Kohn-Sham band structure is obtained from eq 4.

The precise runflow requires the calculation of many magnitudes that depend on integrals on the Brillouin zone, as well as in sums over reciprocal-lattice vectors. These have to be, in practice, cut off to finite limits, imposing a convergence study of the computed solution. The convergence is done in two steps: first, the exchange self-energy is converged, and, then, the correlation part. All convergence tests are done using a 4 × 4 × 1 Monkhorst–Pack sampling of the reciprocal lattice, and at the last step, the variation with the sampling choice is studied. The convergence with respect to the sampling of the Brillouin zone sampling is crucial, due to the localized nature of excitons, but also the most computationally expensive step.

#### Convergence of the exchange self-energy

We start by computing the exchange part of the self-energy from eq 6. The sum on bands *m* accounts for all occupied states and therefore its limits are imposed. The reciprocal space sum ∑ _**G**_ goes to infinite. Then, the gap energy is compared for different levels of convergence. In Figure 6 its evolution with the number of vectors included is shown. We observe that after 90 Ry, which corresponds to ∼70 000 vectors. We adopted 80 Ry as the converged value. The final calculations employed the 90 Ry converged exchange self-energy.

#### Convergence of the Correlation Self-Energy

The correlation self-energy from eq 8 is defined from the microscopic dielectric function:

9
$$\epsilon_{GG^{\prime }}^{-1}\left(q,\omega \right)=\delta_{GG^{\prime }}+v_{G}\left(q\right)\chi_{GG^{\prime }}\left(q\right)$$

where *v*_**G**_(**q**) is the Coulomb potential, and *χ*_**GG**′_(**q**) is the susceptibility function, which satisfies a Dyson equation:

10
$$\chi_{GG^{\prime }}\left(q\right)=\chi_{GG^{\prime }}^{0}\left(q\right)+\underset{G_{1}G_{2}}{\sum }\chi_{GG_{1}}^{0}\left(q\right)K_{G_{1}G_{2}}\chi_{G_{2}G^{\prime }}\left(q,\omega \right)$$

with

11
$$\begin{array}{c} \chi_{GG^{\prime }}^{0}=2\underset{c,v}{\sum }\int_{\mathrm{BZ}}\frac{dk}{{\left(2\pi \right)}^{3}}\rho_{cvk}^{*}\left(q,G\right)\rho_{cvk}\left(q,G^{\prime }\right)f_{v,k-q}\\ \cdot \left[\frac{1}{\omega +E_{vk-q}-E_{ck}}-\frac{1}{\omega -E_{vk-q}+E_{ck}}\right] \end{array}$$

The Kernel entering Dyson’s equation is just $K_{G_{1}G_{2}}=v_{G_{1}}\left(q\right)\delta_{G_{1}G_{2}}+V_{G_{1}G_{2}}^{xc}$ . Thus, the convergence of the correlation self-energy depends on the multiple evaluations of the dielectric function for a number of bands entering the sum in eq 11, and a cutoff sum in eq 10.

In Figure 7 the single-shot *GW* gap is shown as a function of the chosen parameters in the computation of the dielectric function. From the chosen pseudopotential, the Fermi level corresponds to the 58th band. We see that including valence bands from the 30th to the Fermi level (Figure 7b), and conduction bands from the 59th to the 80th (Figure 7a) the *GW* gap is converged. These calculations employed the 90 Ry converged exchange self-energy and 100 bands in the ∑ _*m*_ of eq 8.

The overall sum in bands in eq 8 is the next parameter to check in order to obtain a converged *GW* correction. The *GW* gap dependence with its variation is shown in Figure 8, computed with the converged Hartree–Fock exchange, and the dielectric function. This convergence is usually slow, but at 170 bands, the gap is stable at ∼3.95 eV.

Finally, the *GW* band structure is sensitive to the sample of the Brillouin zone. In Figure 9 the *GW* gap is studied against the choice of the Brillouin zone sample, which enters eq 6, 8 and 11. From this, our final calculation employed the 10 × 10 sample, a 90 Ry cutoff in the exchange self-energy, 10 Ry and the bands range 10–80 in the cutoff of the dynamical screening and 170 bands in the correlation self-energy. We are aware that extremely dense **k**-grids are needed when aiming for an optimal numerical accuracy of the exciton binding energy.^33^ Yet, from our convergence tests, we expect that the contribution from an extreme sampling would not deviate from the eV order of magnitude of our binding energy. In addition, to ensure that our sample provides accurate results, a stochastic integration of the screened potential entering the self-energy was employed.^34^ We call this method by RIM*W* (Random Integration Method of the screened potential *W*). The RIM*W* is based on correcting the screened potential at each point of the small Brillouin Zone with a 2D Monte Carlo integration method. From all possible reciprocal lattice vectors in which the correction can be applied, a cutoff needs to be imposed in practice. Figure 10 we show the convergence of the *GW* gap at a 4 × 4 k-grid with respect to the number of lattice vectors in which the self-energy is corrected. This yielded the final *G*_0_ *W*_0_ band structure in shown in Figure 3a, calculated from eq 4.

## Appendix B

### The Bethe-Salpeter Equation

The variation of Green’s function under an external potential acting simultaneously on two particles defines the two-particle correlation function *L*(1,2;3,4):^30^

12
$$L\left(1,2;3,4\right)\equiv \frac{\delta G\left(1,2\right)}{\delta V_{ext}\left(3,4\right)}$$

It satisfies a Bethe-Salpeter (BS) equation (BSE):^35^

13
$$\begin{array}{c} L\left(1,2;1^{\prime }2^{\prime }\right)=L_{0}\left(1,2;1^{\prime },2^{\prime }\right)\\ +\int d\left(3456\right)L_{0}\left(1,4;1^{\prime }3\right)\Xi \left(3,5;4,6\right)L\left(6,2;5,2^{\prime }\right), \end{array}$$

where *L*_0_(1,2;1’,2’) = *G*(1,2’)*G*(2,1’) and $\Xi \left(3,5;4,6\right)=-iv\left(3,6\right)\delta \left(34\right)\delta \left(56\right)+i\frac{\partial \Sigma \left(3,4\right)}{\partial G\left(6,5\right)}$ . The magnitude Ξ is called BS kernel, and it represents the effects of both bare and screened Coulomb interactions. In the Random Phase Approximation (RPA),^36–38^ the dynamical dependence of the screening is neglected, so that *W*(*ω*) ≈ *W*(*ω* = 0). Under the RPA and in the transition basis, where states are represented by coupled electron-hole pairs (*c* **k**_*c*_,*v* **k**_*v*_), the BSE becomes the secular equation of a two-particle Hamiltonian:^39,40^

14
$$H_{\begin{array}{c} vck\\ v^{\prime }c^{\prime }k^{\prime } \end{array}}=\left(E_{ck}-E_{vk}\right)\delta_{vv^{\prime }}\delta_{cc^{\prime }}\delta_{kk^{\prime }}+\left(f_{ck}-f_{vk\prime }\right)\Xi_{\begin{array}{c} vck\\ v\prime c\prime k\prime \end{array}}$$

The first term on the right-hand side is just the difference between conduction and valence energies at some crystal momentum **k**, implying a correct band structure calculation is needed in advance. For it, we will use the *G*_0_ *W*_0_ energies, already converged. The second term is the product of the difference of occupation factors and the BSE kernel $\Xi_{\begin{array}{c} vck\\ v\prime c\prime k\prime \end{array}}\equiv 2V_{\begin{array}{c} vck\\ v\prime c\prime k\prime \end{array}}-W_{\begin{array}{c} vck\\ v\prime c\prime k\prime \end{array}}$ . This last factor accounts for both unscreened Coulomb interaction $V_{\begin{array}{c} vck\\ v\prime c\prime k\prime \end{array}}$ and the screened electron-hole interaction $W_{\begin{array}{c} vck\\ v\prime c\prime k\prime \end{array}}$ , just as in eq 13. They are calculated as follows:

15
$$\begin{array}{c} V_{\begin{array}{c} vck\\ v\prime c\prime k\prime \end{array}}\equiv \frac{1}{\Omega }\underset{G\neq 0}{\sum }v\left(q+G\right)\rho_{cvk}\left(q+G\right)\rho_{v\prime c\prime k\prime }\left(q+G\right)\\ W_{\begin{array}{c} vck\\ v\prime c\prime k\prime \end{array}}\equiv \frac{1}{\Omega }\underset{G,G^{\prime }}{\sum }v\left(q_{W}+G\right)\epsilon_{G,G\prime }^{-1}\left(q_{W}\right)\delta_{q_{W},k-k^{\prime }}\rho_{cc^{\prime }k}\left(q_{W}+G\right)\rho_{v\prime vk-q}\left(q_{W}+G\right) \end{array}$$

where Ω is the unit cell volume and **q**_*W*_ ≡ **k** – **k** ^′^. In comparison with the correlation part of the *G*_0_ *W*_0_ correction in eq 8, here the dielectric matrix is evaluated at 0 frequency, and it is called ″static″. Its eigenvectors are associated with excitons or linear combinations of electron-hole pairs:

16
$$\left|exc\right\rangle =\underset{cvk}{\sum }A_{cvk}\left|cvk\right\rangle$$

between a hole at the band *v* and an electron at the band *v*, both at momentum **k**.

Therefore, the calculation of an excitonic spectrum goes by the diagonalization of the BS Hamiltonian of eq 14. We applied the Tamm-Dancoff approximation (TDA), in which the BS Hamiltonian is block-diagonal in the transition basis.^41,42^ The TDA drastically reduces the computational cost of building and diagonalizing the BS Hamiltonian. However, its validity is argued to depend on the system in which it is applied. For instance, recent reports indicate that the effect of the TDA on **q** = 0 transitions (such as those studied in our work) is minimal.^43^ Other works, however, report strong divergences between the TDA and the full Hamiltonian^44,45^ for confined systems. If the bandgap is sufficiently big, then the effect of neglecting off-diagonal terms should be minimal, and the TDA is a convenient choice. In particular, 2D materials are known to present bigger bandgaps than their bulk counterparts.^46^ In our system, the big *G*_0_ *W*_0_ gap and its intrinsic low dimensionality justifies the use of the TDA.

The BS matrix elements are constructed by a number of conduction and valence bands, entering sums in reciprocal space vectors. Thus, the precise size of the BS Hamiltonian has to be checked in order to provide accurate results. In addition, the screening interaction *W* depends on the static dielectric function $\epsilon_{G,G\prime }^{-1}$ which also has to be properly calculated, as in the calculation of the *G*_0_ *W*_0_ band structure.

Since we are interested in the whole excitonic spectrum, we diagonalized the BS Hamiltonian at different levels of construction, for some number of bands and specific cutoffs in the Kernel. The diagonalization was performed by means of the SLEPc library,^47^ targeting the first 100 eigenvalues, and a 4 × 4 mesh of k-points was employed in the construction of the BSE Kernel. For the final spectrum in Figure 4, the mesh was increased to 10 × 10, after testing the convergence with the sampling of the Brillouin zone, as explained below.

We start then by comparing the spectrum with increasing cutoffs in the dielectric function in Figure 11a. We observe that beyond 10 Ry, the spectrum becomes constant, so we adopted 10 Ry as the converged cutoff of the static dielectric function. Then, in Figure 11b the spectrum evolution with the global cutoff in the reciprocal-space sums of the BS Kernel in eq 15 is shown. We achieved convergence at 10 Ry. Both studies were done with 4 valence bands (55th–58th) and 4 conduction bands (59th–62nd).

Next, the dependence of the excitonic spectrum with the number of bands included in the construction of the BS Hamiltonian is shown in Figure 12. Valence and conduction bands are checked out separately, considering the gap is between the 58th and 59th bands. We conclude that the valence-conduction 30–80 is the converged set-up. For all calculations, a broadening of 0.01 eV was imposed.

Finally, we test the BSE spectra against samplings of the Brillouin zone. Computationally, this is the most demanding step, since every calculation requires a new DFT set of energies, performed at each sampling. We have employed the 10 × 10 *GW* results in the construction of the BSE kernel and the converged parameters from the previous tests. The resulting spectra and binding energies are shown in Figure 13. The tendency shows that increasing up to 18 × 18 × 1 and beyond, the difference in the binding energies of the excitons will be less than 0.05 eV. Hence, our results from Figure 4 have an absolute error of 0.1 eV.

To sum up, in the end, the absorption spectrum converged at 10 Ry of screening cutoff, 10 Ry of BSE cutoff, and 51 BSE bands: from the 30th to the 80th. The final spectrum is in Figure 4, computed on a 10 × 10 × 1 **k**-mesh. The global number of bands imposed in the static dielectric function was 200 bands, which is a safe choice considering the convergence values (around 50 bands) of the dynamical dielectric function at the *GW* level.

## Notes

The 3D convex hull diagram for a single-layer Cu_2_WSe_4_ structure, along with its projections onto the W/Se, W/Cu, and Cu/Se planes and additionally, it contains a plot of the projected densities of states (PDOS), providing insights into the electronic structure of the material (PDF)
