# Supplementary material for: Optical Properties of Single Layer Cu2WSe4 from the Ab Initio Bethe–Salpeter Equation Method
Source: J Phys Chem C Nanomater Interfaces. 2025 Apr 22;129(17):8361–71. doi: 10.1021/acs.jpcc.5c00855 (PMC12128214; doi:10.1021/acs.jpcc.5c00855)
Supplement: Supplementary file 1 [file jp5c00855_si_001.pdf]

Optical properties of single layer Cu_2WSe_4 from ab initio Bethe-Salpeter-Equation method (supporting information)

Tarik Ouahrani,*,†,‡ A. Esquembre Kučukalić,¶ R. M. Boufatah,‡ and Daniel Errandonea*,§

†*École Supérieure en Sciences Appliquées, ESSA-Tlemcen, BB 165 RP Bel Horizon, Tlemcen 13000 , Algeria.*

‡*Laboratoire de Physique Théorique, Université de Tlemcen, BP 119, 13000, Algeria.*

¶*Institute of Materials Science (ICMUV), University of Valencia, Catedrático Beltrán 2, E-46980 Valencia, Spain*

§*Departamento de Física Aplicada - Instituto de Ciencia de Materiales, Matter at High Pressure (MALTA) Consolider Team, Universidad de Valencia, Edificio de Investigación, C/Dr. Moliner 50, Burjassot, 46100, Valencia, Spain*

E-mail: tarik_ouahrani@yahoo.fr; daniel.errandonea@uv.es

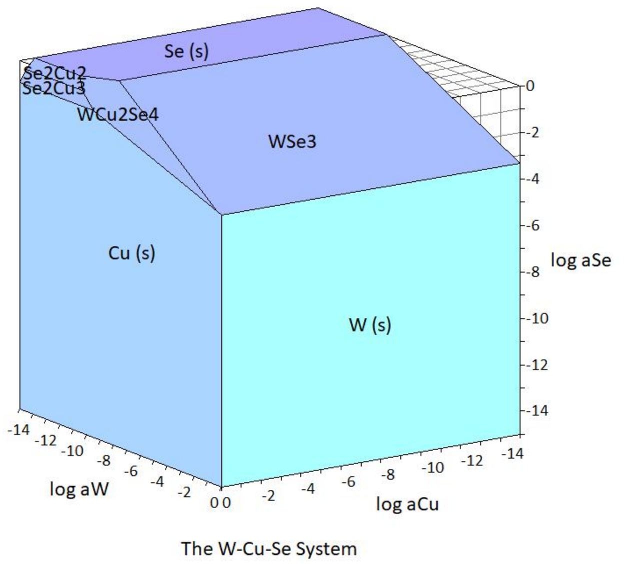

Figure S1 3D convex hull diagram of Cu_2WSe_4 single layer.

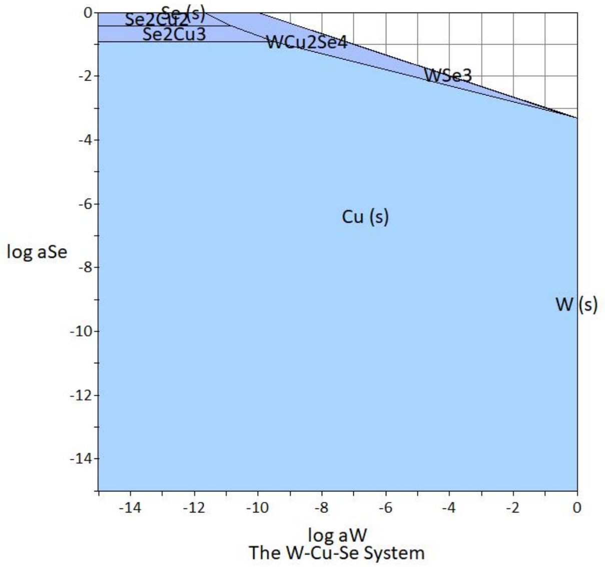

Figure S2 The W/Se projection of the convex hull diagram of Cu_2WSe_4 single layer.

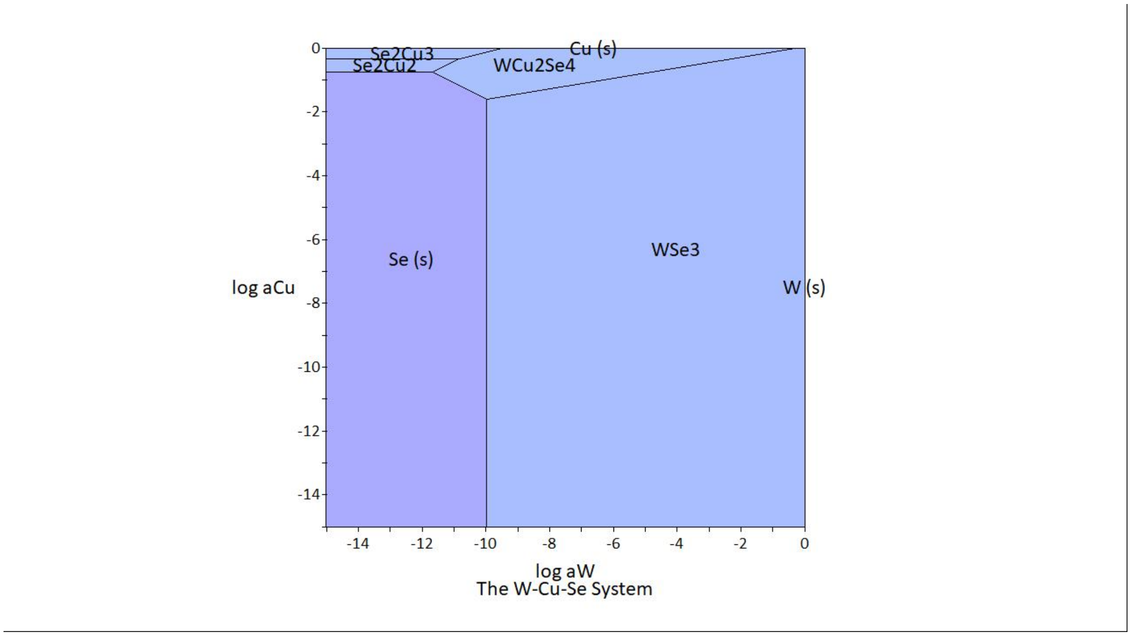

Figure S3 The W/Cu projection of the convex hull diagram of Cu_2WSe_4 single layer.

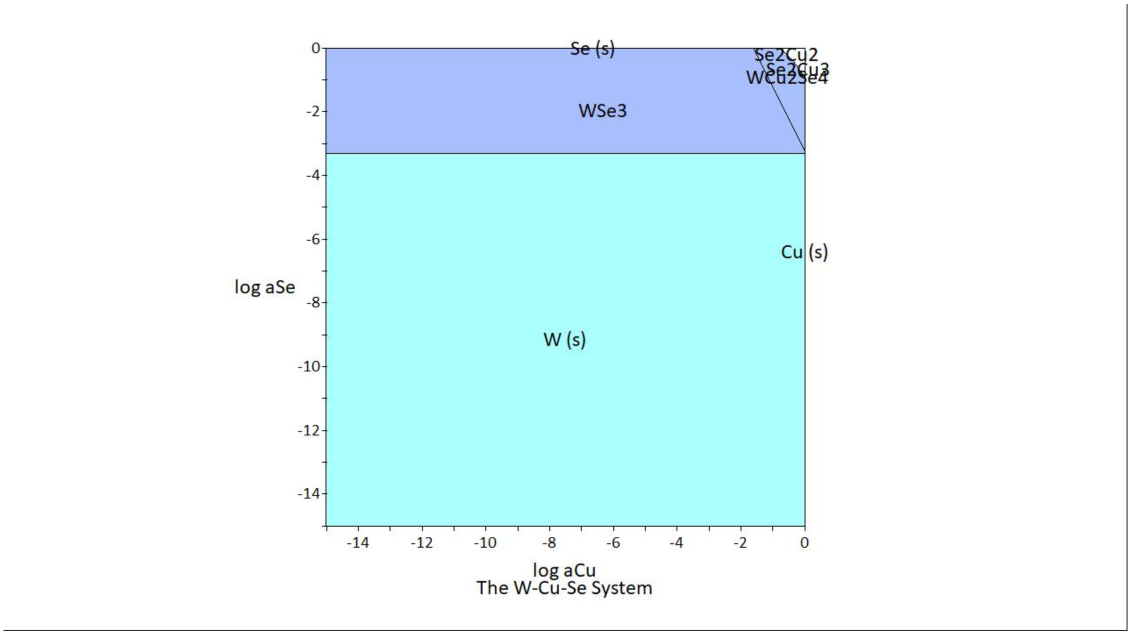

Figure S4 The Cu/Se projection of the convex hull diagram of Cu_2WSe_4 single layer.

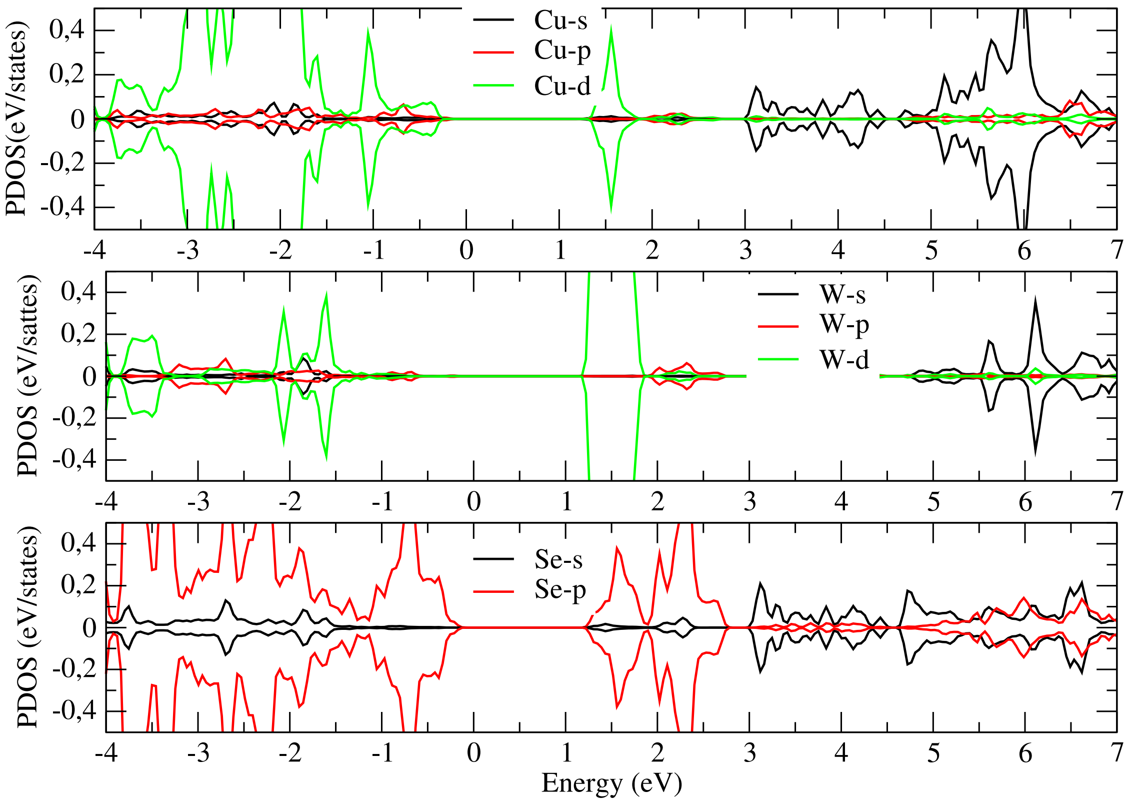

Figure S5 Projected densities of states (PDOS) of Cu_2WSe_4 single layer.
